# Molecular Dynamics Study on the Aggregation Behavior of Triton X Micelles with Different PEO Chain Lengths in Aqueous Solution

**DOI:** 10.3390/molecules28083557

**Published:** 2023-04-18

**Authors:** Jin Peng, Xiaoju Song, Xin Li, Yongkang Jiang, Guokui Liu, Yaoyao Wei, Qiying Xia

**Affiliations:** School of Chemistry and Chemical Engineering, Linyi University, Linyi 276000, China; linqingya1007@163.com (J.P.); 2015110104@lyu.edu.cn (X.S.); lx754362430@126.com (X.L.); justy838@163.com (Y.J.); liuguokui@lyu.edu.cn (G.L.)

**Keywords:** Triton X, micelle, hydration number, solvent accessible surface area

## Abstract

The aggregation structure of Triton X (TX) amphiphilic molecules in aqueous solution plays an important role in determining the various properties and applications of surfactant solutions. In this paper, the properties of micelles formed by TX-5, TX-114, and TX-100 molecules with different poly(ethylene oxide) (PEO) chain lengths in TX series of nonionic surfactants were studied via molecular dynamics (MD) simulation. The structural characteristics of three micelles were analyzed at the molecular level, including the shape and size of micelles, the solvent accessible surface area, the radial distribution function, the micelle configuration, and the hydration numbers. With the increase of PEO chain length, the micelle size and solvent accessible surface area also increase. The distribution probability of the polar head oxygen atoms on the surface of the TX-100 micelle is higher than that in the TX-5 or TX-114 micelle. In particular, the tail quaternary carbon atoms in the hydrophobic region are mainly located at the micelle exterior. For TX-5, TX-114, and TX-100 micelles, the interactions between micelles and water molecules are also quite different. These structures and comparisons at the molecular level contribute to the further understanding of the aggregation and applications of TX series surfactants.

## 1. Introduction

Micelles aggregated from surfactant molecules have been widely used owing to their ability to dissolve hydrophobic compounds effectively. People’s interest in micelle solutions comes from their application potential as functionally molecular assemblies [1]. Different cationic, anionic, zwitterionic, and non-ionic surfactants have been widely studied. Compared with ionic surfactants, non-ionic surfactants are much less toxic and have more efficient surface active properties [2]. Among them, the series of Triton X (TX) non-ionic surfactants have been studied in depth and characterized [3]. Because of their unique molecular structure and amphiphilic nature, the micelles formed by TX surfactants have very flexible surface-active properties. The critical micelle concentration (CMC) of TX surfactant is very low, especially in aqueous solution. Different TX surfactants have been widely used in the fields of microbiology and biomedicine [4].

The TX-100 as one typical nonionic surfactant [5,6,7], has been extensively studied [8,9,10]. This surfactant consists of one hydrophilic chain of 9–10 ethylene oxide units linked to a benzene ring with an octyl chain [6]. Many researchers have explored the structural characteristics of TX-100 micelles in aqueous solution and their interaction with water by experimental techniques such as 2D NOESY NMR [11,12,13], pulsed field gradient NMR [8], solvent paramagnetic relaxation enhancement [7], fluorescence spectra [14,15], surface tension [15], light scattering [16], static and dynamic light scattering [15,17], turbidimetric method [18], small-angle X-ray scattering [13], quasi-elastic light scattering spectroscopy [19]. It is very important to study the hydration and the size of TX-100 micelles [20], because the effectiveness of micellar applications depends on their size and their effects on the properties of the solution [21]. Robson and Dennis [16], Paradies [13], Streletzky and Phillies [19], Phillies et al. [22] used different experimental methods to determine the hydration degree of TX-100 micelles. Other relevant researchers studied the size of the TX-100 micelle by measuring its radius. The aggregation characteristics of TX-100 micelles are also the key aspects that affect their potential applications [7]. Yuan et al. [12] revealed the dependence of the conformation of TX-100 micelles on the environment, and emphasized that the spatial arrangement of PEO chains in micelles is to a certain extent very compact. In addition, the self aggregation and supramolecular micelle structure of TX-100 surfactant molecules in aqueous solution determined by Denkova et al. [11] shows that TX-100 may aggregate in a double-layer or multilayer spherical conformation interlaced between PEO chains and octyl phenyl parts. In order to further explore the structural characteristics, Zhang et al. [7] proposed a more detailed aggregation mode of TX-100 micelles, pointing out that this micelle is more likely to be a multilayer staggered spherical micelle and octyl phenyl is partially dispersed in different layers.

With the development of the computer and algorithm, molecular dynamics (MD) simulation has been widely applied in scientific studies from a micro-perspective. MD simulation is a method of calculating and simulating the time evolution of a group of interacting atoms using Newtonian equations of motion. By solving classical Newton’s equations of motion, the motion trajectory of the system can be obtained. This trajectory includes a large number of samplings of molecular configurations, as well as information of the position and velocity of the particles. Further analysis of the obtained configuration ensemble can provide macroscopic observable properties (density, surface tension, solubility, viscosity, and thermodynamic information) and microscopic properties (intuitive structure, conformational distribution, degree of structural fluctuation, position and intensity of non-bonded interactions, particle spatial distribution) of the system. The description (force field) of the interactions between molecules and atoms is the key for this method to obtain reliable results. This calculation method shows a high degree of relevance in the detailed characterization of self-assembly related systems, including complementarity with experimental data, optimization of experimental design, and prediction of related properties of chemical systems. This may be expensive or difficult for experimentation [23]. Moreover, MD simulations have been effectively applied to investigate the self-assembly and interfacial adsorption of surfactant systems.

In addition to many experimental studies, some literature studies [24,25,26,27,28,29] further discussed the aggregation characteristics of TX-100 micelles at the molecular level by MD simulation. Among these literature studies, the simulation results of several of these literature studies [24,25,28,29] show that the shape and behavior of micelles mainly depend on the aggregation number of aggregates. An important application of TX-100 micelles is that the hydrophobic part can dissolve insoluble organic compounds [30]. A detailed understanding of the pure micelle system is a prerequisite for the study of organic matter and micelle composite systems [31]. In order to better understand this solubilization mechanism, researchers [26,27] explored the basic structure of micelles under a specific aggregation number. Farafonov et al. [26] first observed that the surface of the micelles was highly irregular when they were finally balanced in aqueous solution. Generally, when the temperature was 298 K, the aggregation number of the TX-100 micelle measured by experiments [8,19,22] was about 100. Therefore, Pacheco-Blas and Vicente [27] studied the morphology of TX-100 micelles with an aggregation number of 100 with MD simulation. It was observed that the micelles appeared as a quasi-sphere. The hydrophobic parts of the surfactants preferentially gather in the core of the micelles; however, there is some probability that these hydrophobic groups may be located at the outer region of the micelle.

Micelle structure in surfactant solutions is an important factor affecting the properties and application fields of self-assembly [11]. In this paper, we used all-atom MD simulations to study the self-aggregation properties of TX-5, TX-114, and TX-100 surfactant systems. By analyzing micelle shape and size, the solvent accessible surface area (SASA) of the micelle, the micelle morphology, as well as the interactions between micelles and water molecules, the differences and connections of these three micelles could be compared.

## 2. Results and Discussion

**Figure 1:**
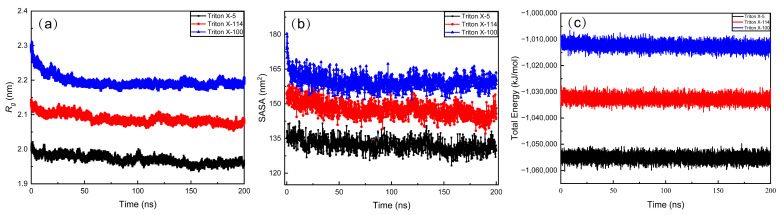
(**a**) The radius of gyration of TX-5, TX-114, and TX-100 micelles with time evolution, (**b**) the solvent accessible surface area of TX-5, TX-114, and TX-100 micelles with time evolution, and (**c**) the total energy of TX-5, TX-114, and TX-100 micelles with time evolution.

Figure 1 shows the time evolutions of radius of gyration (*R*_g_), the solvent accessible surface area (SASA), and the energy of the system during a simulation run to determine the equilibriums of the systems. As can be seen from Figure 1a,b, the *R*_g_ and SASA of three micelles tend to be stable after 150 ns. The total energies of the three systems hardly change during the whole process. These changes of *R*_g_, SASA, and total energies indicated that our simulated systems all reach equilibrium after 150 ns, and the last 30 ns trajectory of each system was used for result analyses in this paper.

### 2.1. Micelle Shape and Size

#### 2.1.1. Micelle Shape

The ratio of the moments of inertia *I*_max_/*I*_min_ is usually used to characterize the shape of the micelle, where *I*_max_ and *I*_min_ are the largest and the smallest moment of inertia along the x, y, or z axis, respectively. When *I*_max_/*I*_min_ is equal to 1, it is a perfect spherical micelle [32]. According to the results obtained in Table 1, the ellipsoidal degree of TX-100 micelle is significantly greater than that of TX-5 and TX-114 micelles. The TX-114 micelle is slightly more spherical than the TX-5 micelle. In addition, eccentricity (*e*) is also an important indicator to judge the shape of the micelle, which is defined as *e* = 1−*I*_min_/*I*_avg_ [32]. The *I*_avg_ is the average moment of inertia of micelle. When *e* is equal to 0, the micelle shows a perfect spherical shape. When micelle shape is stable, the eccentricity will converge near a certain value and small fluctuations will appear [33]. In order to further verify the accuracy of the calculation results, we calculated the *e* values of the three micelles. The *e* of the TX-100 micelle is about 0.12, which is close to the value (0.08) of the TX-100 micelle obtained by Pacheco-Blas et al. [27]. The results show that the ellipsoidal degree of the TX-100 micelle is the largest, and the spheroidal degree of the TX-114 micelle is slightly larger than the TX-5 micelle.

#### 2.1.2. Micelle Size

The size of the micelle is one of the important characteristics of micelle structure, in which *R*_g_ is a standard to characterize the size of the micelle [34]. It can be seen from Table 1 that *R*_g_ increases with the increase of the PEO chain length. In addition, the average micelle radius (*R*_s_) is also used to judge the size of the micelle, and its definition [35] is as follows:

$$R_{s}=\sqrt{\frac{5}{3}}\;R_{g}$$

According to the results obtained in Table 1, the change trend of *R*_s_ is the same as that of *R*_g_. The radius value of the TX-100 micelle (≈2.83 nm) obtained in this paper is consistent with the radius value of the TX-100 micelle (≈2.87 nm) simulated by Pacheco-Blas et al. [27] and the experimental result (3.1 nm) obtained by Brown et al. [8]. Our calculated results are therefore considered to be reliable.

#### 2.1.3. Solvent Accessible Surface Area

Solvent accessible surface area (SASA) is the surface area of the TX micelle in contact with solvent, which can well reflect the properties of the micelle surface. To calculate the SASA of the micelle, the double cubic lattice method [36] was applied. With this method, all water molecules are first removed from the system [35], and then one spherical probe molecule with a radius of 1.4 Å is used to simulate the rolling of water molecules on the surface of the micelle [37]. Table 1 lists the total SASA and the hydrophilic and hydrophobic SASA values of the three micelles. We find that all SASA values increase with the increase of PEO chain length, which is consistent with the increasing trend of micelle size. The hydrophobic surface area of each micelle is much larger than the hydrophilic surface area, and the proportion of the hydrophobic area increases with the rise of the polar head chain. This is on account of the polar head of the research system being polymerized by PEO, and each PEO contains a hydrophobic C_2_H_4_ group and a hydrophilic O group. Therefore, the hydrophobic part accounts for a large proportion. In addition, some hydrophobic tails are distributed outside of the micelle. These hydrophobic groups will also increase the proportion of the hydrophobic surface area of the micelle.

### 2.2. Micelle Structure

**Figure 2:**
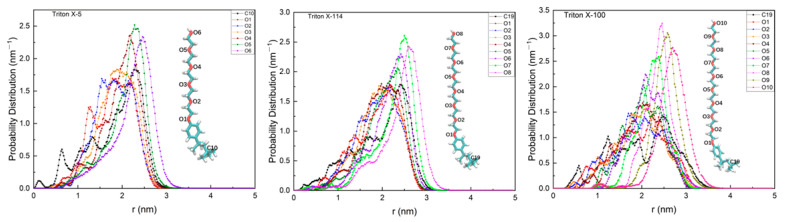
Probability distribution of O atoms at different position and the carbon at the end of the hydrophobic tail chain in TX-5, TX-114, and TX-100 micelle systems with respect to micelle COM.

The spatial distribution of atoms in a micelle can characterize the structural properties of the micelle [38]. The main difference between the non-ionic surfactant molecules of the TX family lies in the number of ethylene oxide units or the chain length [29]. In order to analyze the structures of the three micelles TX-5, TX-114, and TX-100 studied in this paper, we calculated the probability distributions of O atoms at different positions in each micelle system and the carbon atoms at the end of the hydrophobic tail chain relative to the micelle center of mass (COM). All the results are shown in Figure 2.

In general, the nonpolar alkyl chain of amphiphilic surfactant molecules usually aggregates in the hydrophobic region of the micelle, while the polar chain is exposed in the hydrophilic region. Sodium dodecyl sulfate (SDS) is a commonly used ionic surfactant and has been widely studied. Shelley et al. explored the structure of the SDS micelle in aqueous solution by MD simulation. It was found that most of the terminal C atoms of the SDS hydrophobic chain were distributed in the interior of the micelle, and only a small part were distributed on the surface of the micelle [39]. Gao et al. [40], Palazzesi et al. [38], MacKerell et al. [41], and Liu et al. [35] also found a similar phenomenon. It should be noted that these studies are ionic surfactants with small tail chains and polar heads. For TX micelle, there is a relatively large benzene ring near the end of the hydrophobic tail chain and long polar PEO chain head, which is very different from the SDS ionic surfactant. Pacheco-Blas et al. [27] used MD simulation to study the behavior of the non-ionic surfactant Triton X-100 in extracting metal ion Cd^2+^ in an aqueous environment. In the paper, it was found that the hydrophobic chain had a relatively high distribution in the outside of the micelle. In addition, Zhang et al. [7] considered that the TX-100 micelle was more likely to be a multilayer spherical micelle. Molecules from different layers were staggered, and octyl phenyl was dispersed in different layers. In this study, we found that the probability distribution of the tail C of the TX-100 micelle with respect to micelle COM is relatively wide, ranging from the micelle core to the outside of the micelle. In particular, the probability distribution of the terminal C in the outer region of the micelle is higher. The TX-5 and TX-114 micelles also show a similar phenomenon. This may be due to the large benzene rings in the hydrophobic region of the micelles, which are spatially exclusive and widely distributed. Although the tail C has a high probability in the outer region of the micelle, its distribution is also located on the left side of the distribution of the outermost polar O atoms. This means that most tail C atoms are included by the external polar O atoms, to avoid contact with environmental water as much as possible. The distributions of both hydrophobic terminal C and polar head O atoms are multimodal, indicating the existence of the micelle multilayer structure.

**Figure 3:**
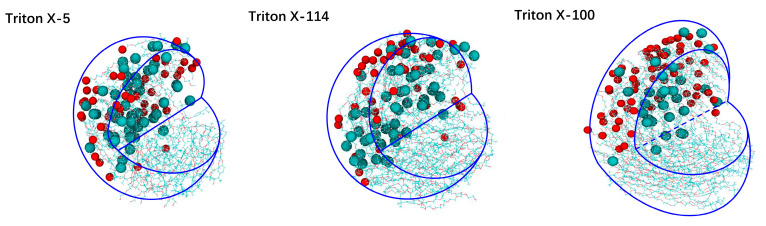
Representative structures of TX-5, TX-114 and TX-100 micelles. Note: the dark green ball in the figure symbolizes C atom, the red ball symbolizes O atom, and the blue auxiliary line symbolizes the outline.

In order to show the differences among the three micelle structures intuitively, we highlighted the C atom at the end of each micelle and the outermost O atom on the polar head to observe their internal structures more clearly. It can be seen from Figure 3 that the micelle shape is consistent with that obtained by the ratio of the moment of inertia and the eccentricity. In agreement with the multimodal probability distributions of terminal C atoms from the micelle center to the micelle surface, some terminal C atoms of the hydrophobic chains are distributed widely in the whole micelle.

### 2.3. Interactions between Micelle and Water

#### 2.3.1. Hydration Numbers

**Figure 4:**
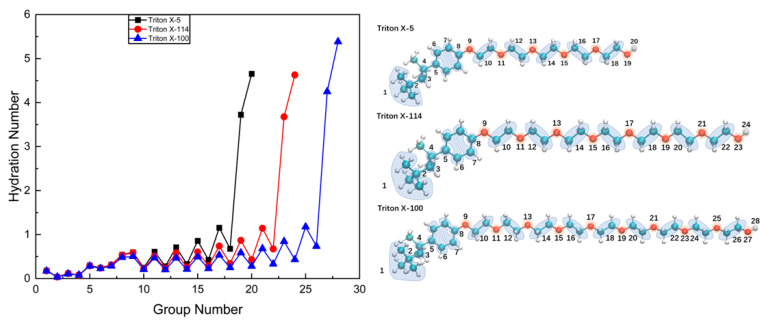
Hydration numbers of C and O atoms at different positions in TX-5, TX-114, and TX-100 surfactant molecules. Note: the Triton X molecular diagram on the right describes the atoms corresponding to each number. Dark green sphere represents C atoms, red sphere represents O atoms, and gray-white sphere represents H atoms.

The interactions between the micelle and water are characterized by calculating the hydration numbers of C and O atoms at different positions in TX-5, TX-114, and TX-100 surfactant molecule. For convenient analysis, the selected C and O atoms are grouped. Among them, a C atom with a similar chemical environment was selected and averaged for calculation, as shown in Figure 4. The hydration numbers were obtained by calculating the radial distribution function (RDF) integral of water molecules around the selected C atom or O atom in the range of 0.35 nm [40,42]. It can be seen from Figure 4 that the three systems have a similar distribution. From the end of the hydrophobic chain to the position of the polar head, the hydration numbers of the selected atoms show increasing trends. The hydration numbers of C atoms 1–4 on the hydrophobic chain are very low. The hydration numbers of C atoms in the benzene ring increase slightly. For C atoms and O atoms in the PEO chain, the hydration numbers present a zigzag trend. The hydration number of the O atom in each ethylene oxide (EO) unit is greater than that of the C atom. Moreover, the hydration numbers of the O atom closer to the position of the polar head is larger. The hydration numbers of the outermost O atoms of the polar head are significantly larger than those of other atoms (>3) with the decreased sequence of TX-100 > TX-114 ≈ TX-5. The hydrations numbers of H atoms on the OH groups also show this sequence. In addition, the hydration numbers of the C atoms at the same position in the three micelles are similar.

#### 2.3.2. Number of Hydrogen Bonds

**Figure 5:**
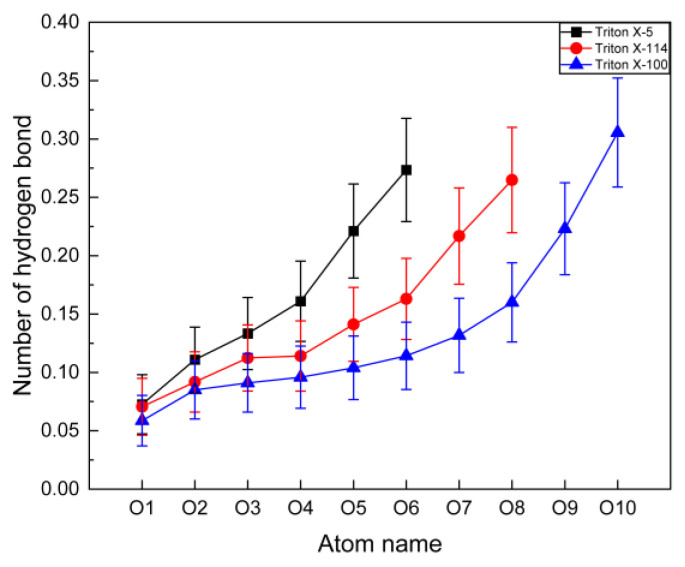
The average numbers of hydrogen bonds formed between O atoms at different positions and water molecules in a surfactant molecule in TX-5, TX-114, and TX-100 micelles. The O atom label is same as the schematic diagram of Figure 2.

In order to further characterize the interactions between the three micelles and water, we calculated the average numbers of hydrogen bonds between the O atoms at different positions and the water molecules. The geometric criterion for the existence of hydrogen bonds is that the distance between donor and acceptor pairs is within 3.5 Å and the angle between hydroxyl and hydrogen atoms is less than 120° [43]. As shown in Figure 5, the numbers of hydrogen bonds of the three systems show a significant upward trend from inner O atom to micelle surface O atom. Among them, the numbers of hydrogen bonds formed between the O atom close to the hydrophobic moiety and the water molecules are the lowest. In previous discussions, the hydration numbers of the outmost polar head O atoms of the three systems were the largest, ca. 4. However, the number of hydrogen bonds formed between these O atoms and water molecules is ca. 0.3, which means that not all the hydrated water molecules can form hydrogen bonds.

#### 2.3.3. Time Correlation Function of the Hydrogen Bond

One method to analyze the dynamic properties of the hydrogen bond is to calculate the intermittent hydrogen bond time correlation function (C_HB_(t)), which is defined as follows:

$$C_{\mathrm{HB}}\left(t\right)=\frac{\langle h(t)h(0)\rangle }{\left(h\right)}$$

This function is considered to be the criterion for judging the formation or fracture of hydrogen bonds, and C_HB_(t) allows the hydrogen bonds to break and reform within the time interval t [44]. The faster C_HB_(t) decays, the more unstable is the hydrogen bond [45].

**Figure 6:**
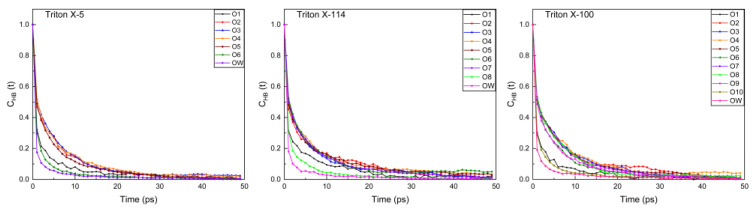
Time correlation function between O atoms at different positions and water molecules, and between the outermost hydroxyl group and O atoms in water molecules in TX-5, TX-114, and TX-100 micelle systems. The O atom label is the same as the schematic diagram of Figure 2. Note: OW represents the O atom in a water molecule.

In this paper, the C_HB_(t) changes of the hydrogen bonds between O atoms at different positions and water molecules and between the outermost hydroxyl group and O atoms of water molecules in the three systems were calculated by this function. As shown in Figure 6, TX-5, TX-114, and TX-100 systems all show three fast decay curves for O atoms connected to hydrophobic chain, the outermost O and H atoms of the polar head. Among these three curves, the outermost H atoms decay fastest. The hydration numbers of the outermost H atoms are the largest and they have a spatial advantage, so that the water molecules that form hydrogen bonds with them exchange faster. This may result in the faster decay curve of the hydrogen bond. The outmost O atoms also behave the same. For internal O atoms, they may behave due to the environmental impact of being connected to the benzene ring of the hydrophobic chain and the spatial limits.

## 3. Materials and Methods

In this paper, we chose the aggregation number of TX-5, TX-114, and TX-100 micelles as 100 according to the experimental values [46,47]. The molecular structures of TX-5, TX-114, and TX-100 were optimized by Gaussian 09 software [48] at the 6-31g(d) level. Then, the force field parameters of all studied molecules were obtained through the automated force field topology builder (ATB) [49,50]. With Packmol software [51], 100 pre-aggregated surfactant molecules for the three micelles were constructed. Then, the TX-5, TX-114, and TX-100 systems were treated with Gromacs 2019 software [52]. Pre-constructed micelle was placed in the center of a 10 × 10 × 10 nm^3^ cube box for each system by inbuilt tools. With the gmx solvate tool, water molecules were randomly added to fill the box. This inbuilt tool can ensure that water molecules are added in the box at locations larger than the volume of water molecules. Subsequently, the tension of the system can be released through pre-equilibrium processes. The simulated systems can achieve equilibrium and obtain a stable conformation. The single point charge (SPC) model was selected to describe water molecules. Specific compositions of all the studied systems are shown in Table 2. After minimizing the energy of the system, a reasonable initial structure was obtained for each system. With the V-rescale method [53], the 2 ns NVT equilibrium was performed to reach the required 298 K. The constant τ_T_ of 0.1 ps was used. Then, the NPT equilibrium with 2 ns was carried out to stabilize the system at 1 bar with a Berendsen pressure controller [54]. In this step, a constant τ_p_ of 2.0 ps was applied. After balancing the temperature and pressure of the system, the 200 ns NVT simulations were carried out to obtain the MD production trajectories. The same temperature controlling method as the NVT equilibrium step was used in the MD production step. In the process of simulation, periodic boundary conditions were used in all directions of the simulation system. The LINCS algorithm [55] was used to limit hydrogen-included bonds, and the particle-mesh Ewald (PME) [56] summation method was used for electrostatic interactions. The 1.4 nm cutoff value was used for van der Waals interactions and short-range electrostatic interactions. The integration time step was 2 fs. For every 10 ps interval, the MD sampling configurations were stored.

## 4. Conclusions

Through MD simulations, we studied the structural characteristics of TX-5, TX-114, and TX-100 micelles at the same aggregation number and for the related properties of each micelle in aqueous solution. With the increase of the PEO chain length, the degree of micelle sphericity was reduced while the micelle radius increased. It is worth noting that the tail C atoms of each micelle were distributed widely with multimodal distributions extending from the micelle COM to the micelle surface. Large parts of the hydrophobic tail of the C atoms were located in the outer region of the micelle. Some polar O atoms close to the hydrophobic chains also showed multimodal distributions. These distributions indicate the existence of a multilayer structure of the TX micelles. The distributions of the tail chains and the polar heads of these three micelles are significantly different from those of traditional cationic and anionic surfactant micelles. For TX-5, TX-114, and TX-100 micelles, the outermost O and H atoms have the largest hydration numbers and hydrogen bond numbers, but the decay of the hydrogen bonds is the fastest. The outermost OH groups of TX-100 show stronger interactions with the surrounding water molecules than those of TX-5 and TX-114. More importantly, the representative structures of TX-5, TX-114, and TX-100 micelles can serve as the structural basis for understanding the properties of the relevant micelles. This study provides a reference for further expanding the exploration and application of TX-5, TX-114, and TX-100 micelles in related fields and it is of great significance for the exploration of other non-ionic surfactant micelle systems.

## Figures and Tables

**Table 1:** Structural characteristics of micelles Triton X-5, Triton X-114, Triton X-100.

	TX-5	TX-114	TX-100
*I_max_/I_min_*	1.15 ± 0.03	1.12 ± 0.04	1.26 ± 0.03
*e*	0.07 ± 0.02	0.06 ± 0.02	0.12 ± 0.02
*R_g_* (nm)	1.96 ± 0.01	2.07 ± 0.01	2.19 ± 0.01
*R_s_* (nm)	2.53 ± 0.01	2.67 ± 0.01	2.83 ± 0.01
SASA(total) (nm^2^)	132.24 ± 2.77	147.52 ± 3.25	159.54 ± 2.97
SASA(hydrophilic) (nm^2^)	30.62 ± 2.33	32.72 ± 2.27	35.81 ± 1.96
SASA(hydrophobic) (nm^2^)	101.66 ± 2.19	114.77 ± 2.29	123.71 ± 2.54

**Table 2:** Specific composition of micelle system Triton X-5, Triton X-114, Triton X-100.

Molecule	TX-5 (*n* = 5)	TX-114 (*n* = 7)	TX-100 (*n* = 9)
N_agg_	100	100	100
H_2_O	30,830	30,264	29,751

*n* represents oxyethylene groups.

## Data Availability

The data presented will be made available on request by the corresponding authors.
